# Immunocytochemistry and fluorescence imaging efficiently identify individual neurons with CRISPR/Cas9-mediated gene disruption in primary cortical cultures

**DOI:** 10.1186/s12868-017-0377-9

**Published:** 2017-08-01

**Authors:** Hiroto Tsunematsu, Akiko Uyeda, Nobuhiko Yamamoto, Noriyuki Sugo

**Affiliations:** Cellular and Molecular Neurobiology, Graduate School of Frontier Biosciences, Osaka University, 1-3 Yamadaoka, Suita, Osaka, 565-0871 Japan

**Keywords:** CREB, FOS, CRISPR/Cas9, Cortical neuron, Gene disruption, Immunocytochemistry

## Abstract

**Background:**

CRISPR/Cas9 system is a powerful method to investigate the role of genes by introducing a mutation selectively and efficiently to specific genome positions in cell and animal lines. However, in primary neuron cultures, this method is affected by the issue that the effectiveness of CRISPR/Cas9 is different in each neuron. Here, we report an easy, quick and reliable method to identify mutants induced by the CRISPR/Cas9 system at a single neuron level, using immunocytochemistry (ICC) and fluorescence imaging.

**Results:**

Dissociated cortical cells were transfected with CRISPR/Cas9 plasmids targeting the transcription factor cAMP-response element binding protein (CREB). Fluorescence ICC with CREB antibody and quantitative analysis of fluorescence intensity demonstrated that CREB expression disappeared in a fraction of the transfected neurons. The downstream FOS expression was also decreased in accordance with suppressed CREB expression. Moreover, dendritic arborization was decreased in the transfected neurons which lacked CREB immunoreactivity.

**Conclusions:**

Detection of protein expression is efficient to identify individual postmitotic neurons with CRISPR/Cas9-mediated gene disruption in primary cortical cultures. The present method composed of CRISPR/Cas9 system, ICC and fluorescence imaging is applicable to study the function of various genes at a single-neuron level.

## Background

Gene manipulation is an indispensable method to understand neuronal functions in the brain. Gene targeting is the most advanced method to disrupt specific gene function [1]. Generation of knockout mice through genetically manipulated ES cells is a standard approach, but requires a lot of time and effort before obtaining results [2, 3]. The recently developed clustered regularly interspaced short palindromic repeat (CRISPR)/CRISPR-associated 9 (Cas9) system enables induction of DNA double-strand breaks selectively to specific genome positions [4–6]. Such DNA damage frequently causes deletions and insertions in the target position. Indeed, this method enables rapid generation of mutant cell and animal lines [4–7]. Moreover, it has also been applied to disruption of gene function in postmitotic neurons [8–11]. In these studies, it is necessary to validate the gene disruption in individual cells, as the effects of CRISPR/Cas9 are distinct between transfected neurons [8–11]. For this, DNA sequence analysis is a direct approach to reveal a mutation in the expected genome position, but it is not realistic to confirm mutations in every single cell.

In the present study, we developed a method using immunocytochemistry (ICC) and fluorescence imaging to identify mutants generated by the CRISPR/Cas9 system in primary neuron cultures. The effectiveness of this method was tested in mouse cultured cortical neurons, focusing on gene disruption of the activity-dependent transcription factor c-AMP responsive element binding protein (CREB) [12]. The CRISPR/Cas9 vector targeting *Creb* was transfected into mouse cortical neurons, and CREB expression level was examined quantitatively using ICC with a specific antibody and fluorescence imaging. We further investigated CREB downstream gene expression at a single-neuron level. Finally, we studied the influence of CREB disruption on dendrite arborization of cortical neurons.

## Methods

### Animals

ICR mice were used (Japan SLC). Noon of the day on which the vaginal plug was detected in the morning was designated embryonic day (E) 0.

### Plasmids

A neuron-specific βIII tubulin promoter-driven EGFP expression vector (pTα1-EGFP) was used to label neurons in primary dissociated neuron cultures [13]. pX330-U6-Chimeric BB-CBh-hSpCas9 (hereafter referred to as CRISPR/Cas9 vector) was purchased from Addgene (plasmid ID: 42230). CRISPR Design tool (https://www.atum.bio/eCommerce/cas9/input) was used to select a single-guide RNA (sgRNA) targeting mouse *Creb* [14]. The candidate sequences were checked by BLAST search (https://blast.ncbi.nlm.nih.gov/) to minimize the off-target activities. To generate the CRISPR/Cas9 vector targeting *Creb*, the annealed oligonucleotide corresponding to the sgRNA (sense: 5′-CACCGGACTTATCTTCTGATGCACC-3′, anti-sense: 5′-AAACGGTGCATCAGAAGATAAGTCC-3′) was introduced into *Bbs*I site in the vector. A plasmid vector pFN21AB5414 containing HaloTag-human CREB1 cDNA was purchased from Promega. To generate the CRISPR/Cas9 resistant CREB expression vector, a deletion mutation of Gly-147 was introduced into pFN21AB5414 by PCR-mediated site-directed mutagenesis with the mutagenic primer pairs: 5′-TGCACCAGTGCCAAGGATTGAAGAA-3′, 5′-CTTGGCACTGGTGCATCAGAAGATAA-3′.

### Primary cortical neuron culture and pharmacological treatment

Pregnant mice were deeply anesthetized with pentobarbital (50 mg/kg, i.p.). Cortical lobes were dissected from E16 mouse embryos in ice-cold Hanks’ balanced salt solution and minced with fine scissors. The minced tissues were incubated with 0.125% trypsin and 0.02% EDTA in PBS for 5 min and dissociated thoroughly by pipetting. After a brief centrifugation, the cells were resuspended in DMEM/F12 medium (Life Technologies) supplemented with B27 (Life Technologies) and 5% fetal bovine serum (Hyclone). Aliquots of the cell suspension containing 1–1.5 × 10^5^ cells were plated with culture medium on a 12 mm circular cover glass in a 4-well culture dish (Nunc, Thermo Scientific), which had been coated with 0.1 mg/ml poly-l-ornithine (Sigma). The cultures were maintained at 37 °C in an environment of 5% CO_2_ and humidified in 95% air. To depolarize the cultured cells, 0.41 volumes of KCl depolarization solution (170 mM KCl, 1.3 mM MgCl_2_, 0.9 mM CaCl_2_, 10 mM HEPES, pH 7.4) was added to the culture medium.

### Transfection

Cultured cortical neurons were transfected with the plasmids by electroporation at 1 day in vitro (DIV). Plasmid DNA in Opti-MEM (0.5 µg/µl, Life Technologies) was added to the culture dish, and electric pulses were delivered with plate electrodes (LF513-5, BEX, Japan) connected to a square-pulse generator (CUY21EX, BEX). One 275 V pulse of 10 ms duration and ten 30 V pulses of 50 ms duration were applied at 50 ms intervals. After the electroporation, the plasmid solution was replaced with the culture medium described above. Neuro2a cells were transfected with the plasmids by Lipofectamine 2000 (Life Technologies) following the manufacturer’s procedure.

### Surveyor assay

After the transfection, Neuro2a cells were plated on 100 mm cell culture dish (100 cells/dish) followed by ~10 days incubation to form colonies. Twenty-four colonies were randomly picked up, and were replated in a 48-well cell culture plate after trypsinization. After several days of incubation, isolated clones were expanded to a 24-well cell culture plate.

Genomic DNA was extracted using MagExtractor Genome (TOYOBO) following the manufacturer’s procedure. Genomic loci including CRISPR/Cas9 targeted site were amplified by PCR using KOD-plus-neo PCR enzyme (TOYOBO) with the primers 5′-GCAGGCACCAGGCATGTGCAG-3′ and 5′-ACAGGCTGGCAAGCCAACATCAT-3′. Amplification (655 bp) was confirmed by agarose gel electrophoresis. The PCR products (1–2 μg) were diluted with 20 μl dilution buffer (20 mM NaCl, 10 mM Tris–HCl, pH 8.0). Then the DNA solution was subjected to denaturing (95 °C, 2 min) and re-annealing (RT, 1 h) to form a heteroduplex. These products were treated with T7 endonuclease I (New England Biolabs) at 37 °C for 1 h. The digested DNA fragments were analyzed by agarose gel electrophoresis. The gels were stained by ethidium bromide and imaged by LAS-3000 mini (Fujifilm) with a UV transilluminator.

### Single-cell genomic PCR

Individual EGFP-labeled neurons were aspirated to a glass capillary-pipette under a fluorescent microscope with a small volume of 50 mM Tris–HCl (pH 8.0). The pipette contents were ejected into 9 μl Proteinase K solution (1.0 mg/ml, Nacalai Tesque) in a 0.2 ml thin-walled amplification tube and then incubated at 55 °C for 15 min followed by 75 °C for 20 min. Nested PCR was used to amplify the locus of CRISPR/Cas9 targeted site in *Creb*. The first-round amplification was performed in a mixture of the DNA solution, 10 μl of Tks Gflex DNA Polymerase Low DNA (2×) (R091A, Takara Bio) and 1 μl of primers (5 μM) 5′-GCAGGCACCAGGCATGTGCAG-3′ and 5′-ACAGGCTGGCAAGCCAACATCAT-3′. The first-round PCR program consisted of one cycle of DNA denaturation at 95 °C for 2 min, followed by 50 cycles of 95 °C for 10 s, 60 °C for 30 s, 68 °C for 1 min. The second-round amplification was performed in a 20 μl volume including 2 μl template DNA from the first-round of amplification, 10 μl of Tks Gflex DNA Polymerase Low DNA (2×) and 1 μl of nested primers (5 μM) 5′-TTATACTGCCCCACCACCAC-3′ and 5′-GCAAACAATATTGGCAGCAA-3′. The second round PCR program consisted of one cycle of DNA denaturation at 95 °C for 2 min, followed by 50 cycles of 95 °C for 10 s, 55 °C for 30 s, 68 °C for 1 min. The second-round PCR product was cloned by TA cloning using pGEM-T Easy vector system (A1360, Promega) according to the manufacture’s instruction. DNA sequencing was performed with the BigDye Terminator v3.1 Cycle Sequencing Kits (Thermo Fisher Scientific) and Applied Biosystems 3130xl DNA analyzer.

### Immunocytochemistry

Cortical neurons were fixed at room temperature for 10 min in 4% paraformaldehyde/PBS. They were then permeabilized and blocked for 15 min in buffer G, composed of 5% normal goat serum (Vector Labs) and 0.1% Triton X-100 in PBS. The cells were then incubated overnight at 4 °C with the primary antibody in buffer G. The antibodies used are as follows: rabbit polyclonal anti-CREB antibody (GeneTex, 1:100), mouse monoclonal anti-CREB antibody (86B10, Cell Signal Technology, 1:100), rabbit monoclonal anti-c-Fos antibody (9F6, Cell Signal Technology, 1:200) and rat monoclonal anti-GFP antibody (GF090R, Nacalai Tesque, 1:1000). For visualization, the cultures were further incubated at RT for 2 h in buffer G containing the following secondary antibodies: Alexa 488-conjugated anti-rat IgG (A11006, Life Technologies, 1:400), Cy3-conjugated anti-mouse IgG (AP192C, Millipore, 1:200), Cy3-conjugated anti-rabbit IgG (AP182C, Millipore, 1:400) or Cy5-conjugated anti-rabbit IgG (711-175-166, Jackson ImmunoResearch, 1:200). Nuclei were stained with 0.1% 4′,6-diamidino-2-phenylindole (DAPI, Sigma) in a mounting medium containing 50% glycerol and 2.3% 1,4-diazabicyclo[2.2.2]octane (Sigma) in 50 mM Tris–HCl (pH 8.0).

### Imaging analysis

All images were captured with an epi-fluorescence microscope (Axiophoto with 20×/0.5 or 40×/0.75 objective lens, Carl Zeiss; Ti-E with 20×/0.75 or 40×/0.95 objective lens, Nikon) attached with a CCD camera (DP70, Olympus; CoolSNAP HQ2, Photometrics) or an EM-CCD camera (iXon897, Andor Technology). Fluorescence intensities of CREB and FOS expressions were quantified using ImageJ software. The background fluorescence intensities in the cytoplasm, which lack these transcription factors, were subtracted from the fluorescence intensities in the nucleus, and then the expression levels were determined by the ratio of the subtracted nuclear intensities to the cytoplasmic intensities. Measurements of dendrite morphology were performed using NeuronJ plugin [15].

### Statistical Analysis

All statistical values are presented as the mean value ± SEM from at least three independent experiments. Significant differences were determined with Mann–Whitney’s *U* test and Kolmogrov–Smirnov (KS) test. Excel (Microsoft) was used for statistical analysis and data plotting.

## Results

### Vector construction for targeted gene disruption using CRISPR/Cas9 system

**Fig. 1:**
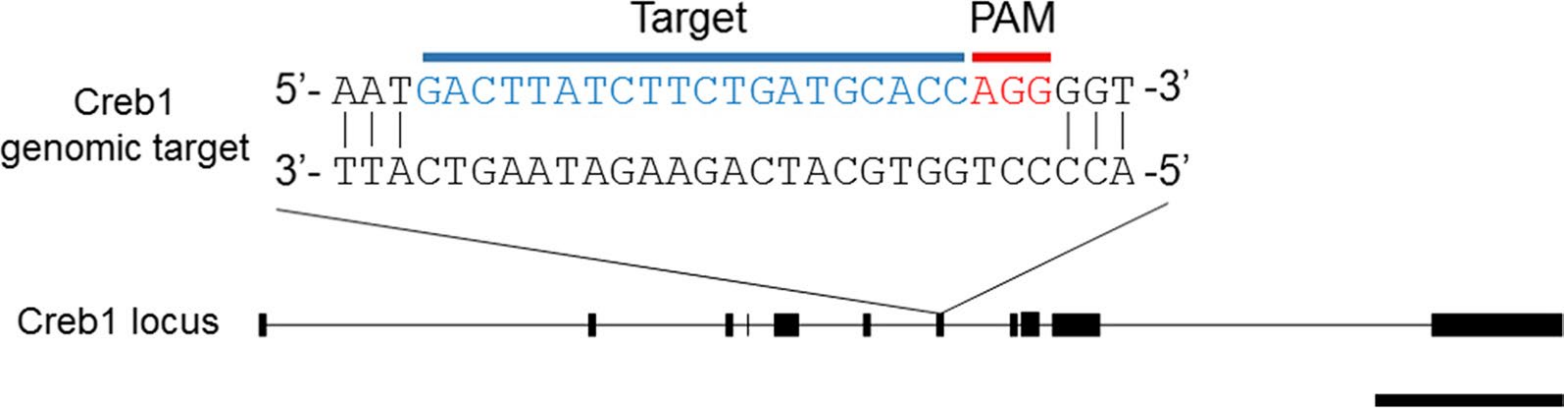
Graphical representation of the mouse *Creb* and the CRISPR/Cas9 target site. The targeted genome sequence (20 bp, *blue*) and the PAM sequence (*red*) are represented with *Creb* locus. The sgRNA targets Cas9 to the exon 7 of *Creb*. *Scale bar* 10 kbp

To disrupt CREB function in mouse cortical neuron cultures using the CRISPR/Cas9 system, the sgRNA, which guides Cas9-endonuclease, was designed by using a web-based search tool for finding 20 nucleotides followed by a 5′-NGG, the requisite protospacer-adjacent motif (PAM) sequence, in exons of the *Creb* gene (see “Methods”). In this study, we selected the sgRNA sequence targeting exon 7 of *Creb* from several candidates (Fig. 1), because exon 7 is included in the major isoforms [16]. The annealed oligonucleotide corresponding to the sgRNA sequence was inserted into the CRISPR/Cas9 vector, expressing both sgRNA and Cas9-endonuclease in mammalian cells [4].

### Targeted gene disruption in Neuro2a cells using CRISPR/Cas9 system

**Fig. 2:**
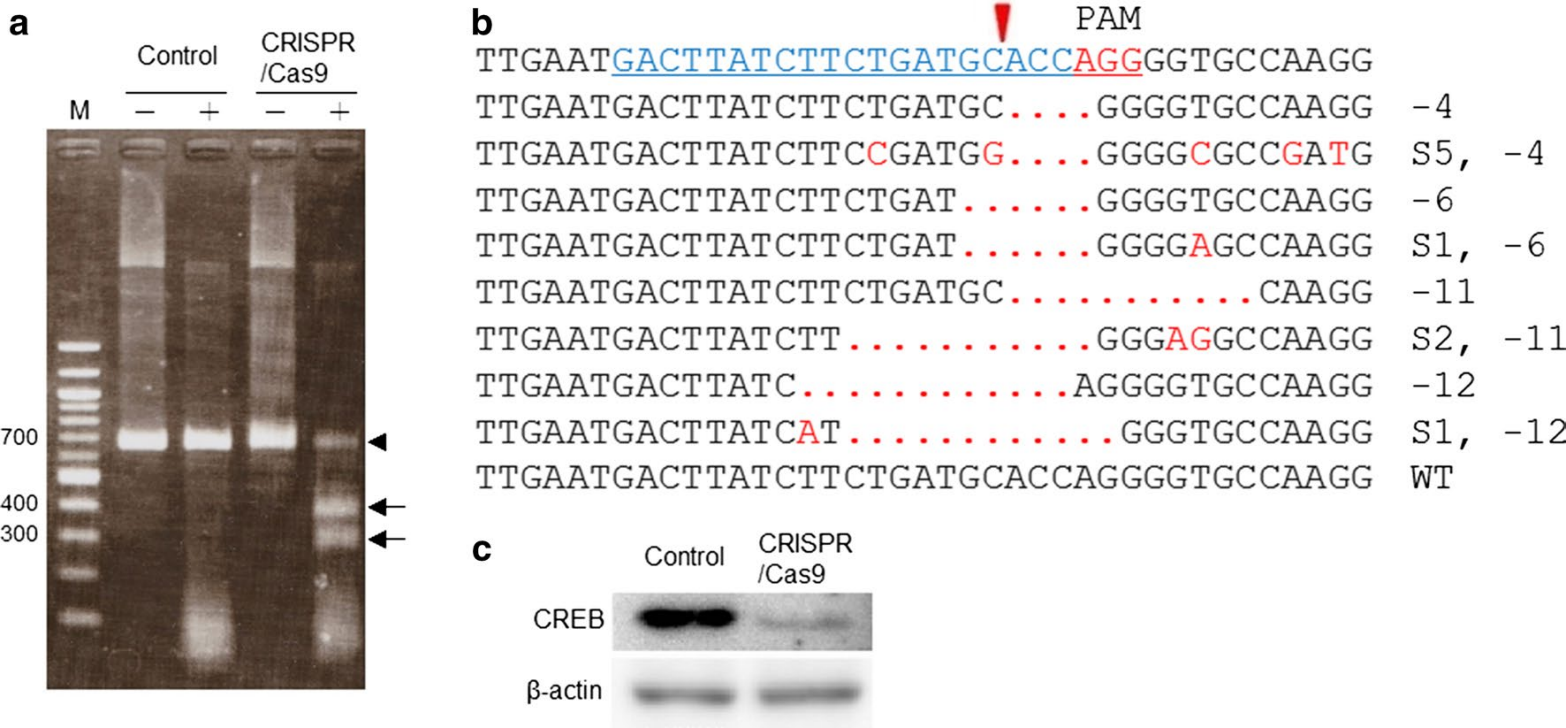
The CRISPR/Cas9 induces mutations in *Creb*. **a** Agarose gel electrophoresis shows the result of the Surveyor assay in the targeted *Creb* loci of control Neuro2a cells (control) and the cloned CRISPR/Cas9 vector transfected cells (CRISPR/Cas9). *Plus and minus signs above each lane* indicate the presence and absence of T7 endonuclease I, respectively. An *arrowhead* indicates the PCR product (655 bp). *Two arrows* indicate the digested fragments of the PCR product by T7 endonuclease I. **b** Representative mutation patterns revealed by DNA sequencing of the target site in the exon 7 of *Creb*. *Top* indicates the targeted sequence (*blue underline*) and PAM sequence (*red underline*) in *Creb*. An *arrowhead* indicates the Cas9 cut site. *Red dots* indicate deleted bases. *Red characters* indicate base substitutions. The number of deletions (−) and base substitutions (S) are shown. **c** Western blot analysis was performed with anti-CREB and β-actin antibodies. The lysates were prepared form controls (control) and the cloned transfected cells (CRISPR/Cas9)

To examine the ability of the plasmid vector encoding CRISPR/Cas9 targeting *Creb*, we studied CREB expression in the transfected Neuro2a cells. After colony formation following Lipofectamine 2000-mediated transfection, several clones were randomly picked up and analyzed using the Surveyor assay [17]. The Surveyor assay represented a mutation within *Creb* in the cloned cells (Fig. 2a). Subsequently, DNA sequencing analysis showed a variety of mutations, including deletion and base changing, in the expected site of *Creb*, although we also identified unchanged wild-type sequences in the cloned cells (Fig. 2b). Because the variety of mutations identified was more than the expected number of target sites in the Neuro2a genome, the mutations may have been introduced at several times during proliferation of the cloned cells. Indeed, western blot analysis revealed substantially decreased but not completely eliminated CREB protein expression in the cloned cells (Fig. 2c). A similar result has been reported in the case of CRISPR/Cas9 mediated mouse and rat mutagenesis showing mosaic mutations in founder animals [18, 19]. Taken together, these results indicate that the CRISPR/Cas9 vector enables to introduce frameshift mutations into *Creb* in mouse genome.

### Targeted gene disruption in primary dissociated cortical neurons using CRISPR/Cas9 system

**Fig. 3:**
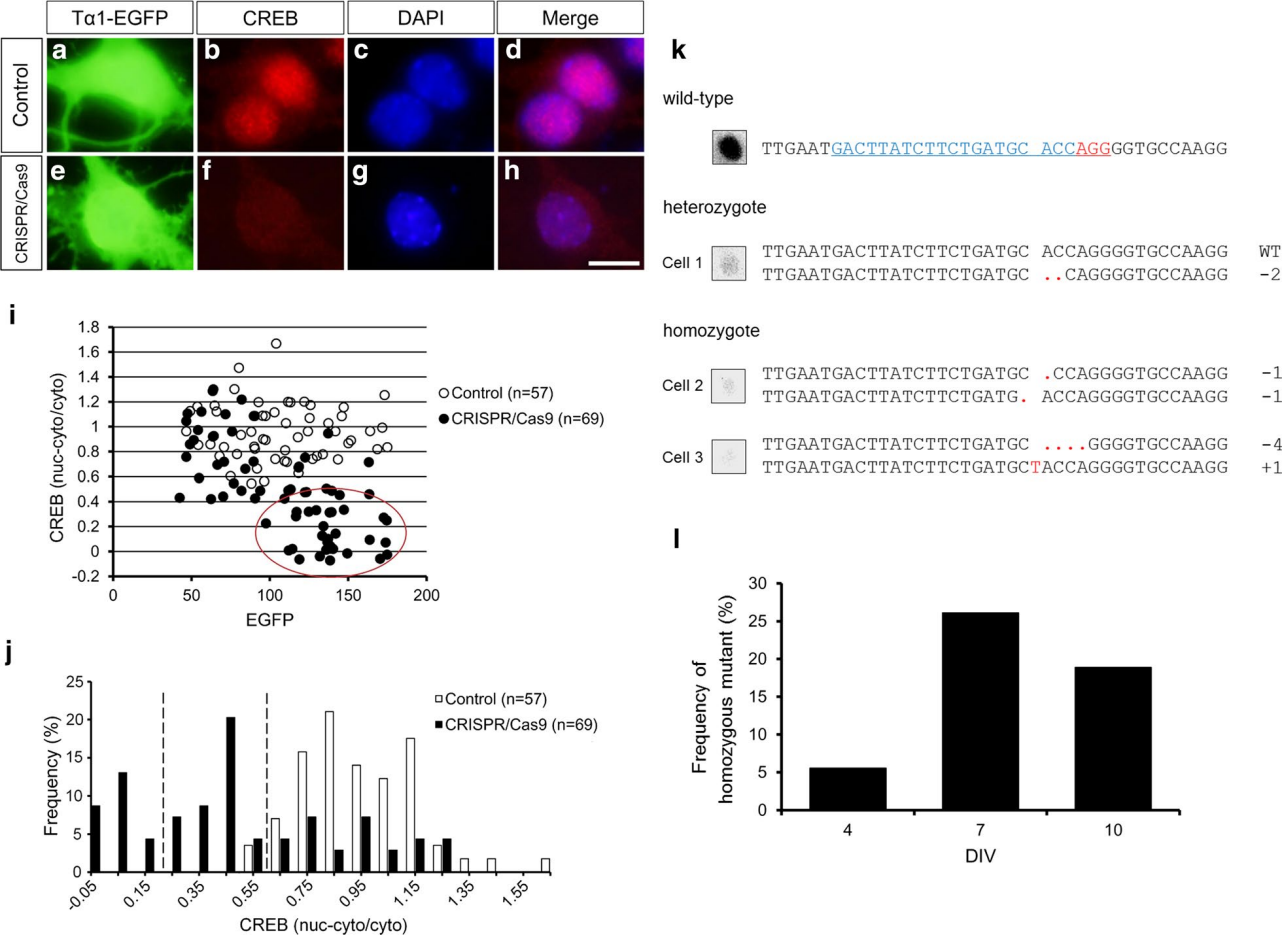
Quantitative analysis of CREB expression in the CRISPR/Cas9-transfected cortical neurons. Cultured cortical neurons were transfected with pTα1-EGFP (control, **a**–**d**) or the CRISPR/Cas9 vector together with pTα1-EGFP (CRISPR/Cas9, **e**–**h**). The cultures were fixed after 6 days following the transfection. ICC analysis was performed with multi-color fluorescence labeling of EGFP (Alexa488, *green* **a**, **e**), CREB (Cy3, *red* **b**, **f**) and DAPI (*blue* **c**, **g**). *Scale bar* 10 μm. **i** Scatter diagram shows the immunofluorescence intensities of EGFP and CREB expression in the neurons co-transfected with CRISPR/Cas9 vector and pTα1-EGFP (CRISPR/Cas9, *black*) and pTα1-EGFP transfected neurons (control, *white*) at 7 DIV. A *red circle* indicates the neurons with strong EGFP and weak CREB expression. **j** Distribution histogram shows the CREB expression level in the CRISPR/Cas9-transfected neurons (*black*) and the control neurons (*white*) at 7 DIV. The difference between the two distributions is significant (KS test, *P* < 0.001). *Interrupted vertical lines* indicate boundaries of the estimated fractions which correspond to the very low and the moderate expression of CREB in the CRISPR/Cas9-transfected neurons. **k** Representative mutation patterns of the target site in the exon 7 of *Creb* revealed by single-cell genome PCR and DNA sequencing in the CRISPR/Cas9-transfected neurons. ICC analysis was performed with anti-CREB antibody (*left side images*). *Top* indicates the targeted sequence (*blue underline*) and PAM sequence (*red underline*) in wild-type *Creb* locus. *Red dots* indicate deleted bases. The number of deletions (−) and insertions (+) are shown.** l** Histogram shows the frequency of the homozygous mutant neurons in the CRISPR/Cas9-transfected neurons in different culture periods. (4 DIV, n = 36 cells; 7 DIV, n = 69 cells; 10 DIV, n = 53 cells)

To investigate the effect of the CRISPR/Cas9 vector targeting *Creb* in dissociated cortical neurons, pTα1-EGFP was transfected with or without the CRISPR/Cas9 vector. Instead of DNA sequencing analysis as a method for the genotyping of *Creb*, we directly examined an expression level of CREB protein in individual transfected neurons using ICC with anti-EGFP (Alexa488, green), anti-CREB (Cy3, red) antibodies and DAPI (blue) after fixation at 7 DIV. Control EGFP-positive neurons which were transfected with pTα1-EGFP alone showed strong CREB accumulation in the nuclei (Fig. 3a–d). In contrast, CREB expression in the nucleus almost disappeared in a fraction of the co-transfected neurons (Fig. 3e–h). To examine whether the different expression levels were caused by either heterozygous or homozygous *Creb* mutation, the fluorescence intensities of CREB were quantitatively examined in individual EGFP-labeled neurons. To quantify the intensity of immunostaining strictly, we performed ICC simultaneously for the control and the CRISPR/Cas9 transfected cultures. Then, CREB expression level was determined by the ratio of nuclear to cytoplasmic fluorescence intensities (see “Methods”). First, we noticed that CREB expression level tended to decrease in the high EGFP-expressing neurons, suggesting that the amount of transfected plasmids affects the frequency of targeted gene disruption (Fig. 3i). As shown in Fig. 3j, the distribution of CREB expression levels had a single peak in the control EGFP-positive neurons. In contrast, the distribution of the expression levels in the CRISPR/Cas9 transfected neurons showed two extra peaks in lower expression levels, suggesting that these fractions are due to the heterozygous and the homozygous CREB mutations (Fig. 3j). The fraction expected to contain the homozygous mutants was defined as very low (the signal intensity was <18% compared to the mean of the controls). To confirm the estimated genotypes, the sequence of the cleavage site of *Creb* locus was examined in several transfected neurons by using single-cell genomic PCR. The results indicated that the mutations were detected in one or both alleles roughly corresponding to the protein expression levels of CREB (Fig. 3k). The homozygous mutations were frequently observed in the neurons with very low expression level of CREB (the signal intensity was <18% compared to the mean of the surrounding controls; 4/4 neurons). These results suggest that a method using ICC and fluorescence imaging can be applied to identify homozygous mutants generated by the CRISPR/Cas9 system in primary neuron cultures.

### Longer culture period increases frequency of the homozygous *Creb* mutation

Transient gene expression in postmitotic neurons is thought to persist for a longer period than that in mitotic cells, because cell division dilutes the transfected plasmids. We examined the possibility whether a longer culture period after the transfection increases the frequency of gene disruption (Fig. 3l). The estimated fraction of the homozygous mutant neurons was increased between 4 DIV (5.6%) and 7 DIV (26.1%), but it was retained at 10 DIV (18.9%). This result suggests that the transfected CRISPR/Cas9 vector continues to work at least for 7 days in postmitotic neurons.

### Analysis of CREB function in the CRISPR/Cas9-transfected neurons

**Fig. 4:**
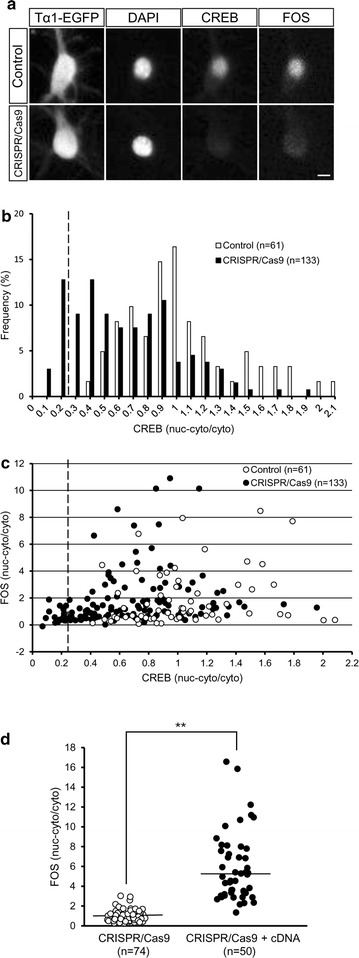
FOS expression in CREB-deficient cortical neurons after KCl treatment. **a** Cultured cortical neurons were transfected with either pTα1-EGFP alone (control) or the CRISPR/Cas9 vector together with pTα1-EGFP (CRISPR/Cas9). The cultures were fixed at 7 DIV after 2 h KCl treatment. ICC was performed with multi-color fluorescence labeling of EGFP (Alexa488), CREB (Cy3), FOS (Cy5, *far red*) and DAPI. *Scale bar* 10 μm. **b** Distribution histogram shows the CREB expression level in the CRISPR/Cas9-transfected neurons (*black*) and the control neurons (*white*). The distribution of the CRISPR/Cas9-transfected neurons is significantly different from the control neurons (KS test, *P* < 0.001). An *interrupted vertical line* indicates a boundary between the estimated fraction of the homozygous mutant neurons and others. **c** *Scatter diagram* shows the expression of FOS and CREB in the neurons transfected with CRISPR/Cas9 vector and pTα1-EGFP (CRISPR/Cas9, *black*) and pTα1-EGFP transfected neurons (control, *white*) at 7 DIV. An *interrupted vertical line* indicates a boundary between the estimated fraction of the homozygous mutant neurons and others. **d** *Dot plots* show the FOS expression level in the CRISPR/Cas9-transfected neurons (control, *white*) and the CRISPR/Cas9-resistant rescue *Creb* cDNA and the CRISPR/Cas9-cotransfected neurons (*black*). The number of neurons analyzed in each case is presented in *parentheses*. *Bars* represent the mean. *Asterisks* indicate a significant difference from control cells (Mann–Whitney’s *U* test, **P < 0.01)

CREB is known to promote downstream *Fos* gene expression directly in response to neuronal activity [20]. If the CRISPR/Cas9 disrupted CREB function, activity-dependent FOS expression level would be decreased. Although CREB knockout mice exhibit decreased FOS expression in the cerebral cortex [21, 22], a quantitative relationship between CREB and FOS expression levels still remains unclear at a single neuron level. We carefully examined activity-dependent FOS expression in the CRISPR/Cas9-transfected cortical neurons using ICC analysis with multi-color fluorescence labeling of EGFP (Alexa488), CREB (Cy3), FOS (Cy5, far red) and DAPI. The CRISPR/Cas9-transfected neurons were treated with high KCl medium at 7 DIV when spontaneous neuronal activity is still low [23]. After 2 h incubation, FOS expression substantially decreased in a fraction of the CRISPR/Cas9-transfected neurons (Fig. 4a, c). The fraction firmly associated with decreased FOS expression was approximately consistent with the fraction of the homozygous CREB mutant neurons (15.8%, Fig. 4b, c), suggesting that the lack of CREB expression inhibits FOS induction. To ascertain this causal relationship, we also performed a rescue experiment with the CRISPR/Cas9 resistant *Creb* cDNA expression vector plasmids. The rescue vector was co-transfected to cortical neuron cultures followed by examination of FOS expression levels. The neurons with extremely low expression levels of FOS disappeared in the co-transfected neurons, but not in the CRISPR/Cas9-tranfected neurons (Fig. 4d). Thus, the CRISPR/Cas9 system, ICC and imaging analysis revealed that CREB regulates activity-dependent FOS expression at a single-neuron level.

**Fig. 5:**
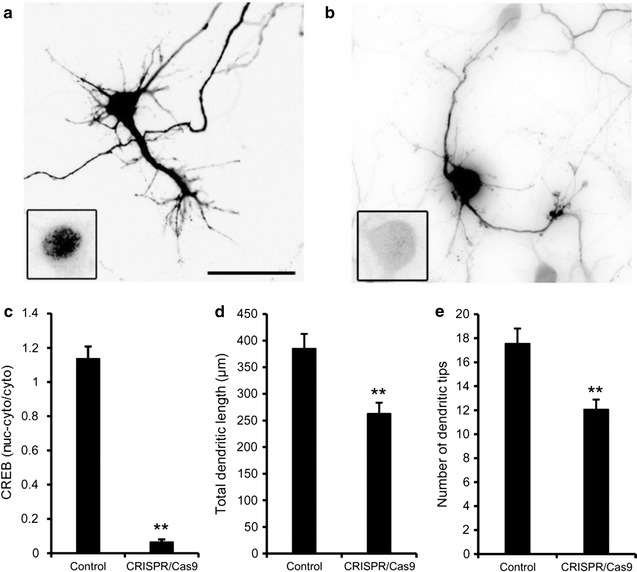
Dendrite formation in CREB-deficient cortical neurons. Cultured cortical neurons were transfected with either pTα1-EGFP (control, **a**) or the CRISPR/Cas9 vector together with pTα1-EGFP (CRISPR/Cas9, **b**). The cultures were treated with high KCl medium for 2 h at 7 DIV and fixed. The *insets* are enlarged images of cell bodies stained with anti-CREB antibody. *Scale bar* 50 μm. Quantitative analysis was carried out for CREB expression (**c**), dendrite length (**d**) and the number of dendrite tips (**e**). *Bars* represent the mean ± SEM (control, n = 25 cells; CRISPR/Cas9, n = 31 cells). *Asterisks* indicate a significant difference from control cells (Mann–Whitney’s *U* test; **P < 0.01)

CREB-mediated gene expression is thought to regulate dendrite morphology in cortical neurons [12]. Although the dominant negative mutant of CREB has shown to diminish dendrite growth in cortical neurons [24], genetic evidence is still insufficient. To address this issue, we quantitatively analyzed dendrite morphology of cortical neurons in which the expression level of CREB was greatly decreased by CRISPR/Cas9 (Fig. 5a–c). We found that the total dendrite length and the number of dendrites were significantly decreased in the homozygous CREB mutant neurons (total dendritic length: 263.1 ± 20.4 μm, n = 31, *P* < 0.01; the number of dendritic tips: 12.1 ± 0.8, n = 31, *P* < 0.01) compared to CREB-expressing neurons (385.2 ± 27.5 μm, n = 25; 17.6 ± 1.3, n = 25; Fig. 5d, e). These results support that CREB-mediated gene expression is required for dendrite growth in cortical neurons.

## Discussion

In the present study, we investigated CREB function in individual postmitotic cortical neurons by developing a CRISPR/Cas9-based method combined with ICC and fluorescence imaging. This was a very quick and convenient technique to analyze gene function in individual neurons.

An advantage in the present technique is that ICC and imaging analysis are much easier to estimate the homozygous gene mutation than sequence analysis at a single-cell level, although a strict criterion is necessary to identify homozygous mutants precisely (Fig. 3j). However, if this method is applied to other genes, it may be necessary to determine optimal conditions since immunoreactivity could be different in each antibody. On the other hand, the quantitative analysis demonstrated the relationship between CREB and FOS expression in individual cortical cells transfected with the CRISPR/Cas9 vector (Fig. 4), although the frequency of target gene disruption in this study seems slightly lower than that in previous studies [8–11]. Thus, even when the transfection efficiency is not necessarily high, the present technique brings benefits to the investigation of gene function in postmitotic neurons, which is comparable to the electrophysiological analysis combined with CRISPR/Cas9 system [8, 9].

RNA interference (RNAi) mediated knockdown using siRNA and shRNA is an established method to investigate gene function in neuronal cultures [25]. Our present method using CRISPR/Cas9-mediated gene disruption may provide a more definite and stable phenotype caused by the homozygous mutation than the RNAi knockdown at a single-neuron level (Figs. 4, 5). Instead, a longer period seems to be required for the complete disruption of gene function. In fact, CREB expression was not eliminated at 4 DIV after the transfection (Fig. 3l). Co-transfection of in vitro synthesized Cas9 mRNA or purified protein with sgRNA may improve the delay in cultured neurons [7, 18, 26, 27].

We found that FOS expression and dendrite growth were suppressed in the CREB-deficient cortical neurons (Figs. 4, 5). This is consistent with the previous result that dendrite formation was decreased by the dominant negative mutant of CREB [24], and further suggests that FOS promotes dendrite formation in CREB downstream. Down-regulated FOS expression (Fig. 4) may lead to the decrease of dendrite growth, because FOS protein directly binds to enhancer sites of the genes which regulate dendrite formation such as histone deacetylase Hdac9 [23, 28]. Thus, our method confirms CREB-mediated dendrite formation in developing cortical neurons.

## Conclusion

ICC and imaging analysis efficiently identifies individual neurons with CRISPR/Cas9-mediated gene disruption, and reveals changes of downstream gene expression and morphological defects. This combined method is certainly applicable to various studies which explore specific gene function in the developing brain.

## Abbreviations

CREB: cAMP-response element binding protein
CRISPR/Cas9: clustered regularly interspaced short palindromic repeat/CRISPR-associated 9
ICC: immunocytochemistry
PAM: requisite protospacer-adjacent motif
sgRNA: single-guide RNA
